# Influence of preeclampsia and gestational obesity in maternal and newborn levels of vitamin D

**DOI:** 10.1186/s12884-015-0547-7

**Published:** 2015-05-13

**Authors:** Homero Rabelo Pena, Marilia Carvalho de Lima, Katia Galeão Brandt, Margarida Maria Castro de Antunes, Giselia Alves Pontes da Silva

**Affiliations:** Hospital Barão de Lucena, Recife, Pernambuco, CEP 52050-041 Brazil; Department of Maternal and Child Health, Universidade Federal de Pernambuco, Recife, Brazil

**Keywords:** Vitamin D, Vitamin D deficiency, Preeclampsia, Obesity, Pregnancy, Newborn

## Abstract

**Background:**

In recent years, a high prevalence of vitamin D deficiency amongst pregnant women and newborns has been observed throughout several regions of the world, especially in the presence of preeclampsia (PE) or obesity (OB). The aim of this study was to investigate whether nonobese and obese preeclamptic pregnant women and their newborns have low 25(OH)D compared with nonobese and obese nonpreeclamptic pregnant women; and to verify whether the maternal level of this vitamin correlates with the newborns’ level.

**Methods:**

This is a cross-sectional study conducted with 179 pregnant women recruited immediately before delivery, divided into four groups: PE^+^/OB^−^; PE^+^/OB^+^; PE^−^/OB^+^; and PE^−^/OB^−^, with gestational age ≥ 34 weeks. Maternal peripheral blood and newborns umbilical cord blood were collected and 25(OH)D levels were measured by chemiluminescence (LIAISON®).

**Results:**

Infants born to preeclamptic mothers had a lower median 25(OH)D level than those born to nonpreeclamptic mothers (*p* < 0.01). Obese pregnant women and their newborns had higher frequencies of 25(OH)D deficiency, but the difference with respect to nonobese pregnant women and their newborns was not significant. The vitamin D status of preeclamptic obese women was not worse than that of their nonobese counterparts. Newborns and maternal 25(OH)D levels were significantly correlated (*p* = 0.01). Obesity weakened this correlation.

**Conclusions:**

Preeclamptic women and their newborns presented higher frequencies of 25(OH)D deficiency, but 25(OH)D levels were not significantly influenced by obesity. Obese pregnant women transferred less 25(OH)D to their fetuses.

## Background

Vitamin D deficiency during pregnancy is common [1, 2] and concerning because of its association with many conditions, such as preeclampsia (PE), obesity, and gestational diabetes, as well as its medium- and long-term repercussions for newborns [1, 3, 4].

Classically, emphasis rests on the importance of vitamin D to bone metabolism [5], but increasingly greater biological and clinical roles have been attributed to its active form 1,25(OH)_2_days in the last years [6]. There has been a paradigm shift such that, today, 1, 25(OH)_2_days is considered a hormone that acts on specific receptors found in at least 36 different types of cells [6].

The participation of 1, 25(OH)_2_days in immune system regulation has been demonstrated [7, 8], so its deficiency is now considered a risk factor for inflammatory diseases, such as PE [8–11]. Prepregnancy obesity is also associated with higher susceptibility to PE [12]. Additionally, studies have shown an inverse relationship between BMI (Body Mass Index) and vitamin D status [13–15]. Hence, low vitamin D in obese pregnant women [16] can contribute to the onset of PE, which is complex and multifactorial [17].

Since fetal vitamin D status depends on maternal levels of this vitamin [1, 18], all situations that cause vitamin D deficiency during pregnancy imply in low vitamin D in the newborn. Bone (craniotabes, low bone mineral content, and rickets) [19] and nonbone repercussions (greater risk of respiratory infections, asthma, and diabetes type 1) [20] have been associated with vitamin D deficiency at birth.

Hence, the objective of this study is to investigate whether nonobese and obese preeclamptic pregnant women and their newborns have low 25(OH)D compared with nonobese and obese nonpreeclamptic pregnant women; and to verify whether the maternal level of this vitamin correlates with the newborns’ level.

## Methods

### Study design and subjects

This cross-sectional study was conducted from November 2012 to March 2013 in the maternity ward of the Hospital Barão de Lucena in the city of Recife, Northeast Brazil. The study was approved by the Human Research Ethics Committee of the Health Sciences Center of the Federal University of Pernambuco and followed the principles of the Declaration of Helsinki. Pregnant women were recruited immediately before delivery after signing the informed consent form. They were divided into four groups: nonobese preeclamptic (PE^+^/OB^−^), obese preeclamptic (PE^+^/OB^+^); obese nonpreeclamptic (PE^−^/OB^+^); and nonobese nonpreeclamptic women (PE^−^/OB^−^).

All subjects had singleton pregnancies with gestational age of at least 34 weeks. They were aged 16 to 40 years and attended the first prenatal care visit before gestational week 16. The exclusion criteria were hypertension before gestational week 20, history of chronic hypertension, gestational diabetes, diabetes mellitus, liver or kidney disease, carrying an infection, or taking anticonvulsants.

Twenty-two of the 201 initially recruited mother-newborn dyads were excluded because of hemolysis or insufficient blood sample (*n* = 16), or inappropriate storage or transportation (*n* = 6), therefore, the study sample comprised 179 mother-newborn dyads.

### Study variables

Gestational age was determined by the date of the last menstrual period. PE was defined as the presence of hypertension (systolic blood pressure level of 140 mmHg or higher or a diastolic blood pressure level of ≥ 90 mmHg) after the twentieth week of gestation in previously normotensive women, plus the presence of proteinuria (≥1+ on dipstick in single urine sample), according to criteria adopted by the American College of Obstetricians and Gynecologists (ACOG) [21]. Maternal nutritional status was assessed by means of BMI obtained in the first prenatal consultation before the 16^th^ gestational week. Women were defined as obese or not according to the classification developed by Atallah *et al.* [22] and adopted by the Ministry of Health of Brazil, which classifies pregnant women according to the appropriateness of BMI in relation to gestational age. No measurements were undertaken to assess women adiposity.

Vitamin D status was classified according to the cutoff points proposed by Holick *et al.* [23] for adults (pregnant women), and the same values were used for the newborns [24]: ≥ 30 ng/mL is considered enough to promote bone and no bone health, 20 to 29.9 ng/mL indicates insufficiency and <20 ng/mL deficiency.

A single researcher used a form to interview the participants after delivery and collect information on socioeconomic and demographic factors, clinical pregnancy data, amount of exposure to sunlight, frequency of sunscreen use, newborns weight and length. Newborns nutritional status was classified according to the appropriateness of weight in relation to gestational age, using the fetal growth curve of Lubchenco *et al.* [25].

### Laboratory analyses

Peripheral and umbilical cord blood samples were collected from the mothers and newborns, respectively, using a Vacuette® tube, which contains clot activator and serum separator gel. The tubes were immediately centrifuged and refrigerated to 2-8 °C until the vitamin D level was determined, no later than 72 h after blood collection. Vitamin D level was determined by chemiluminescence using the commercial assay LIAISON 25 OH Vitamin D Total (310600)®, from DiaSorin Inc. (USA), which has an analytical measurement range of 4.0 to 150.0 ng/mL. All samples were analyzed by a single technician using the same equipment throughout the study period in a reference laboratory. The inter-assay coefficient of variation varied from 6.5 % to 8.1 % during this period, in accordance with the manufacturer’s standards.

### Statistical power and analyses

A retrospective statistical power calculation from the frequencies of vitamin D deficiency observed in PE^+^/OB^−^ and PE^+^/OB^+^ groups in relation to the PE^−^/OB^−^ with a significance level of 5 % showed a sample power around 95 %.

Normality of continuous variables was checked using a histogram. Those with symmetric distribution were expressed as means and standard deviation and the asymmetric ones as medians and inter quartile range (IQR). The associations between categorical variables were tested by the chi-square test. For continuous variables one way analysis of variance (ANOVA) and the Kruskal-Wallis test were employed as appropriate. Pearson correlation coefficient tested the association of two continuous variables. The Statistical Package for the Social Science (SPSS), version 13 was used for the statistical analysis. The significance level was set at 5% for all tests.

## Results

Table 1 shows the socioeconomic and clinical characteristics of the pregnant women and their newborns. Around 63 % (58/92) of the preelamptic women were primigravida as opposed to 31 % (27/87) of the nonpreeclamptic women, and nonobese pregnant women were younger than the obese. There was a greater percentage of small-for-gestational-age (SGA) newborns in the group PE^+^/OB^−^ and a higher frequency of large-for-gestational-age (LGA) newborns in the obese groups (PE^+^/OB^+^ and PE^−^/OB^+^).

**Table 1:** Maternal and newborn characteristics^a^ according to maternal nutritional status and occurrence of preeclampsia

Variables	PE ^+^/OB ^−^	PE ^+^/OB^+^	PE ^−^/OB^+^	PE ^−^/OB^−^	*P*-value
	(n = 48)	(n = 44)	(n = 40)	(n = 47)	
Maternal age (years)	21.0(19.0-27.0)	26.5(21.5-31.5)	27.0(22.0-33.0)	24.0(19.0-29.0)	0.003^*^
Maternal BMI (kg/m^2^)	23.7(21.2-25.7)	32.9(31.5-36.8)	32.8(31.6-35.9)	24.2(21.3-26.3)	<0.001*
Number of pregnancies					
1	70.8% (34)	54.5% (24)	27.5% (11)	34.0% (16)	<0.001^**^
≥2	29.2% (14)	45.5% (20)	72.5% (29)	66.0% (31)	
Gestational age (weeks)					
<37	25.0% (12)	18.2% (08)	12.5% (05)	14.9% (07)	0.43^**^
≥37	75.0% (36)	81.8% (36)	87.5% (35)	85.1% (40)	
Skin color					
White	16.7% (08)	25.0% (11)	17.5% (07)	14.9% (07)	0.62^**^
Other	83.3% (40)	75.0% (33)	82.5% (33)	85.1% (40)	
*Per capita* family income (in Reais)	312(208–500)	339(211–450)	252.00(169–336)	282.70(200–400)	0.09^*^
Literacy					
Illiterate	2.1% (01)	4.5% (02)	10.0% (04)	10.6% (05)	0.28^**^
Literate	97.9% (47)	95.5% (42)	90.0% (36)	89.4% (42)	
Exposure to sunlight (minutes/day)	20.0(10.0-32.5)	20.0(15.0-35.0)	32.5(15.0-50.0)	20.0(10.0-30.0)	0.16^*^
Sunscreen					
Yes	10.4% (05)	09.1% (04)	07.5% (03)	08.5% (04)	0.97^**^
No	89.6% (43)	90.9%(40)	92.5% (37)	91.5% (43)	
Infant sex					
Female	45.8% (22)	59.1% (26)	55.0% (22)	53.2% (25)	0.63**
Male	54.2% (26)	40.9% (18)	45.0% (18)	46.8% (22)	
Birth weight (g)	2782.3 ± 750.9	3272.6 ± 648.6	3326.7 ± 589.3	3221.2 ± 544.3	<0.001***
Birth length (cm)	46.5(44.5-48.0)	48.0(47.0-49.0)	49.0(47.0-50.0)	48.0(46.0-49.0)	0.006*
Nutritional status at birth					
AGA	58.3% (28)	70.5% (31)	75.0% (30)	83.0% (39)	<0.001**
SGA	33.3% (16)	0% (0)	05.0% (02)	04.3% (02)	
LGA	08.4% (04)	29.5% (13)	20.0% (08)	12.7% (06)	

^a^Values are shown as mean ± SD, median (inter quartile range) or %. NB: newborn; AGA: appropriate for gestational age; SGA: small for gestational age; LGA: large for gestational age; OB: obesity; PE: preeclampsia

1.0 Real = US$ 2.0

*Kruskal-Wallis test; **Chi-square test; ***Analysis of variance

Preeclamptic women and their newborns had a higher frequency of 25(OH)D deficiency than nonpreeclamptic women. The differences of vitamin D status between PE^+^/OB^−^ and PE^−^/OB^−^ pregnant women was significant (*p* = 0.0006) as well as between their newborns (*p* = 0.006). The same was observed among the groups of PE^+^/OB^+^ and PE^−^/OB^−^ (*p* = 0.002) and between their newborns (*p* = 0.03). Women and newborns in the group PE^−^/OB^+^ had a higher frequency of 25(OH)D deficiency than those in the group PE^−^/OB^−^, but the difference was not significant (*p* = 0.10 e 0.16, respectively). The vitamin D status of the mother-newborn dyads in group PE^+^/OB^+^ was not worse than that of mother-newborn dyads in group PE^+^/OB^−^ (*p* = 0.62 e 0.72, respectively). (Table 2).

**Table 2:** Distribution of the 25(OH)D status among pregnant women and newborns

25(OH)D	PE ^+^/OB^−^	PE ^+^/ OB^+^	PE ^−^/ OB^+^	PE ^−^/OB^−^	*P*-value^*^
	(*n* = 48)	(*n* = 44)	(*n* = 40)	(*n* = 47)	
Pregnant women					
Adequate	08.3 %(04)	04.6 %(02)	20.0 %(08)	17.0 %(08)	0.002
Insufficient	39.6 %(19)	47.7 %(21)	47.5 %(19)	68.1 %(32)	
Deficient	52.1 %(25)	47.7 %(21)	32.5 %(13)	14.9 %(07)	
Newborns					
Adequate	33.3 %(16)	36.4 %(16)	52.5 %(21)	57.4 %(27)	0.06
Insufficient	52.1 %(25)	54.5 %(24)	40.0 %(16)	42.6 %(20)	
Deficient	14.6 %(07)	09.1 %(04)	07.5 %(03)	0 % (0)	

PE: preeclampsia; OB: obesity; *Chi-square test

**Fig. 1:**
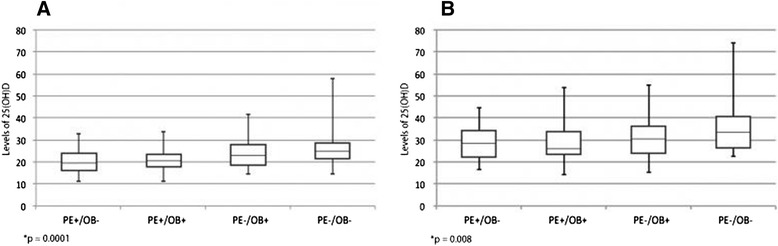
Median, inter quartile range and minimum and maximum values of 25(OH)D in pregnant women (**A**) and newborns – umbilical cord blood (**B**). PE: preeclampsia; OB: obesity. *Kruskal-Wallis test

Preeclamptic women and newborns had a lower median vitamin D level than the nonpreeclamptic women and newborns (Fig. 1). The median differences of vitamin D between PE^+^/OB^−^ and PE^−^/OB^−^ groups were significant among pregnant women (*p* = 0.0002) and among the neonates (*p* = 0.007). The same was observed between the PE^+^/OB^+^ and PE^−^/OB^−^ groups (*p* = 0.0003 for pregnant women and *p* = 0.002 for newborns). However, when analyzing the median differences between PE^−^/OB^+^ and PE^−^/OB^−^ groups we found no statistical significance (*p* = 0.22 among pregnant women and *p* = 0.10 among neonates). The median 25(OH)D level of all newborns was roughly one-third higher than that of all mothers (Fig. 1).

**Fig. 2:**
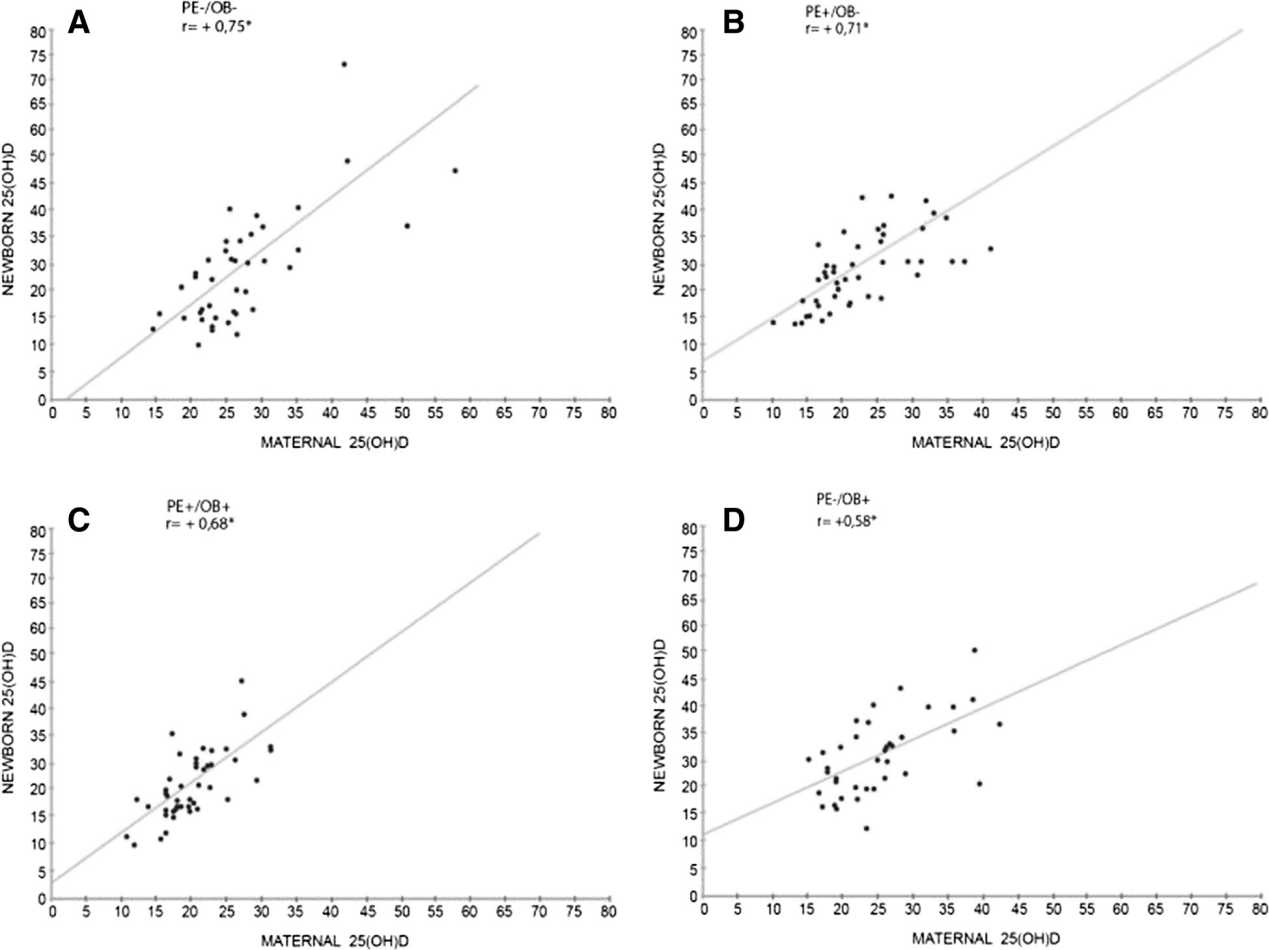
Correlations between maternal and newborn (umbilical cord blood) levels of 25(OH)D. *p = 0.01

Fig. 2 shows that the 25(OH)D levels of nonobese women and their newborns were strongly and positively correlated; the 25(OH)D levels in obese women and their newborns were also positively correlated, but not as much. The correlations in all groups were significant (*p* = 0.01).

## Discussion

Preeclampsia was associated with a lower median vitamin D and a higher frequency of vitamin D deficiency in pregnant women and their newborns. Other studies have also reported worse vitamin D status in preeclamptic women and their newborns [9–11, 26]. Robinson *et al.* [11] and Baker *et al.* [10] found 25(OH)D deficiency percentages of 54% and 26%, respectively, in severely preeclamptic American women, and both percentages significantly exceeded those in nonpreeclamptic women. These studies used the same vitamin D status cutoff points as the present study. In another case–control study, Bodnar *et al.* [9] found a higher percentage of vitamin D deficiency in American women who developed PE than in controls (*p* = 0.08). Likewise, infants born to preeclamptic mothers had significant worse 25(OH)D status than those born to control mothers.

Studies have postulated that maternal vitamin D status affects the risk of preeclampsia [27]. This condition can be partly explained by the manner in which the trophoblast is implanted in the endometrium: an exacerbated maternal immune response (increase of proinflammatory cytokines) allows only superficial implantation, leading to lower maternal tolerance of the fetus [17, 28]. The immunomodulating properties of the active form of vitamin D may be essential during trophoblast implantation, so vitamin D deficiency at the beginning of pregnancy could cause an unbalance between pro- and anti-inflammatory cytokines, constituting a risk factor for PE [29].

Baker *et al.* [10] and Bodnar *et al.* [9] collected blood from pregnant women in the first half of pregnancy and found that low 25(OH)D levels precede clinical PE presentation. Thus, higher causal inference may be speculated, and perhaps low vitamin D levels at the beginning of pregnancy may have had some effect on the complex PE pathophysiology.

The study results show greater vitamin D deficiency in obese women, but the difference was not significant. Bodnar *et al.* [16] also found a higher percentage of mother-NB dyads with 25(OH)D levels below 20 ng/mL when prepregnancy BMI ≥ 30 kg/m^2^ (*p* < 0.05). Low vitamin D levels in obese individuals may stem from this vitamin’s fat-soluble nature: as the percentage of body fat increases, serum 25(OH)D level decreases [13].

Surprisingly, the group PE^+^/OB^+^ did not have worse 25(OH)D status than the group PE^+^/OB^−^. Studies testing serum levels of 25(OH)D in obese preeclamptic women were not found, so comparisons were not possible. Since the present study is observational, selection bias is possible. Most obese participants had BMI from 30 to 35 (grade I obesity), so the results could have been different if there were more women with obesity grades II and III in the group PE^+^/OB^+^. Additionally, the classification of obesity according to BMI only, without analysis of body composition, could have resulted in the selection of pregnant women with a smaller percentage of body fat, implying in smaller sequestration of circulating 25(OH)D [15].

The newborns vitamin D medians were higher than those of the mothers in all groups. In a literature review, Dror & Alen [1] found reports of 25(OH)D levels in cord blood being as much as 108% higher than those of the mothers. The different methods used for measuring serum vitamin D may partly justify the differences. In line with other studies [30, 31], the study newborns levels of vitamin D were positively correlated with those of their mothers during pregnancy, showing that fetuses are dependent on maternal serum 25(OH)D levels. However, according to the coefficients of determination, there was less maternofetal transfer of 25(OH)D in the groups of pregnant women with higher BMI. In a recent study, Josefson *et al.* [32] also found that obese mothers transferred less 25(OH)D to their fetuses than nonobese mothers. Although this fact awaits an explanation, some hypotheses with a nonlinear explanation have been offered, based on the physiological aspects of placental vitamin D transportation and the pathophysiology of obesity.

The mechanism by which vitamin D crosses the placenta is not yet clear. Studies from the 1980s [33] and recent literature reviews [34] report that the main mechanism is passive diffusion, as occurs with sex steroid hormones. The free serum 25(OH)D fraction may reflect the amount available for cells according to the free hormone theory [35]. Therefore, only free 25(OH)D would cross the cellular lipid bilayer because of its solubility in fat. Karlsson *et al.* [36] found that obese women of childbearing age had higher circulating DBP levels than normal weight women, resulting in a smaller percentage of free 25(OH)D. If higher levels of circulating DBP also occur in obese pregnant women, and placental transportation of 25(OH)D is passive, smaller maternofetal transfer of vitamin D is to be expected.

On the other hand, the placenta is a complex organ, and different clinical contexts may change its structure. The first months of pregnancy should promote greater placental expression of the gene CYP27B1, which codifies the enzyme 1α-hydroxylase, necessary for the conversion of calcidiol into calcitriol; and simultaneously silence the gene CYP24A1, responsible for the catabolism of 25(OH)D and 1,25(OH)_2_days [29]. In vitro studies have shown that tumor necrosis factor-alpha (TNF-α) increased CYP24A1 expression in trophoblast cultures [27]. In obese women, the placenta is inflamed, containing high levels of pro-inflammatory cytokines [37]. Therefore, the inflamed placenta of obese women may be an epigenetic factor that increases placental expression of the gene CYP24A1, resulting in lower 25(OH)D levels in the maternofetal interface and consequently, smaller amounts of 25(OH)D transferred to the fetus.

We believe that future studies, especially *in vivo*, can elucidate the mechanism by which vitamin D is transported across the placenta of obese and nonobese women with and without complications, such as preeclampsia, and thereby contribute to a better understanding of newborns’ vitamin D status.

The study results must be analyzed in light of its limitations. Measuring 25(OH)D right before delivery reflects its status only for the past two to three weeks [38]. Moreover, any biomarker has limitations, either because of physiological factors or the methodological difficulties associated with its measurement [35]. The main factors capable of affecting 25(OH)D levels are: higher physiological requirements, body fat, hemodilution and ageing effects, affinity of vitamin D for its target tissues, and cellular absorption efficiency, among others [35]. Although 25(OH)D is the most widely used marker by clinicians and researchers, it only indicates vitamin D supply, so it may not be the best biomarker of biological function [35]. Finally, not having used the gold standard, high-performance liquid chromatography (HPLC), to determine the sample’s vitamin D levels may have returned less accurate 25(OH)D measurements.

It should be emphasized that the present study was conducted close to the equator, at a latitude of 9° south, where UVB radiation is abundant throughout the four seasons of the year. This fact alone controlled this geographic confounder present in studies conducted at high latitudes.

## Conclusion

The study found a smaller median and consequently, worse vitamin D status in mother-newborns dyads with preeclampsia or gestational obesity. Since obesity became one of the greatest global epidemics at the dawn of this century, besides the high prevalence of preeclampsia in pregnancies, we believe that new studies should investigate whether maternal supplementation with higher doses of vitamin D can improve the 25(OH)D status of newborns.

## Abbreviations

1,25(OH)_2_days: 1,25-dihydroxyvitamin D
25(OH)D: 25-hydroxyvitamin D
ACOG: American College of Obstetricians and Gynecologists
ANOVA: Analysis of variance
BMI: Body mass index
DBP: Vitamin D-binding protein
HPLC: High-performance liquid chromatography
IOM: Institute of Medicine
IQR: Inter quartile range
LGA: Large-for-gestational-age
OB: Obesity
PE: Preeclampsia
RDA: Recommended dietary allowance
SGA: Small-for-gestational-age
SPSS: Statistical package for the social science
TNF-α: Tumor necrosis factor-alpha
USA: United States of America
UVB: Ultraviolet B.
